# Distribution of Neuroendocrine Cells and Hormone Receptors in the Male Proximal Urethra: Implications for Sensory Function and the Impact of Aging

**DOI:** 10.1590/S1677-5538.IBJU.2025.0402

**Published:** 2025-09-30

**Authors:** Juan Andrés Venegas, Carlos Gallegos, Paola Ochova, Carlo Lozano, Valentina Pozo, Ivanny Marchant, Pablo Olivero

**Affiliations:** 1 Servicio de Urología Hospital Carlos van Buren Valparaíso Chile Servicio de Urología Hospital Carlos van Buren, Valparaíso, Chile; 2 Universidad de Valparaíso Cátedra de Urología Escuela de Medicina Valparaíso Chile Cátedra de Urología, Escuela de Medicina, Universidad de Valparaíso, Valparaíso, Chile; 3 Servicio de Anatomía Patológica Hospital Carlos van Buren Valparaíso Chile Servicio de Anatomía Patológica Hospital Carlos van Buren, Valparaíso, Chile; 4 Universidad de Valparaíso Laboratorio de Estructura y Función Celular Facultad de Medicina Valparaíso Chile Laboratorio de Estructura y Función Celular, Facultad de Medicina, Universidad de Valparaíso, Valparaíso, Chile; 5 Universidad de Valparaíso Laboratorio de Modelamiento en Medicina Facultad de Medicina Valparaíso Chile Laboratorio de Modelamiento en Medicina, Facultad de Medicina, Universidad de Valparaíso, Valparaíso, Chile; 6 Universidad de Valparaíso Unidad de Estudios Clínicos Facultad de Medicina Valparaíso Chile Unidad de Estudios Clínicos, Facultad de Medicina, Universidad de Valparaíso, Valparaíso, Chile

**Keywords:** Urethra, Estrogens, Neuroendocrine Cells

## Abstract

**Purpose:**

To assess the distribution of neuroendocrine cells in the male penile urethra and the influence of age and estrogen therapy.

**Materials and methods:**

A retrospective immunohistochemical cohort study was conducted on 26 penile urethras obtained during feminizing genitoplasty. Three anatomical regions were analyzed: bulbar, distal, and glans urethra. Neuroendocrine cells were identified by double immunohistochemistry and quantified as chromogranin A-positive cells per mm^2^ of epithelium.

**Results:**

Patients were divided according to estrogen exposure: Group 1 (n=12, median age 23.5 years, estrogen 5.5 years) and Group 2 (n=14, median age 43.0 years, estrogen 18.0 years). A clear gradient of neuroendocrine cell density was observed, with the bulbar urethra showing the highest values compared to distal urethra and glans. Group 2 had significantly higher bulbar urethral cell density than Group 1 (p<0.005). A negative correlation between age and bulbar density was observed.

**Conclusions:**

This study provides evidence of a neuroendocrine cell gradient in the human male urethra, concentrated in the bulbar region. Cell density declines with age but is preserved by long-term estrogen therapy. These findings underscore the bulbar urethra as a key sensory structure that should be preserved during surgery and suggest that hormone therapy may mitigate age-related sensory decline.

## INTRODUCTION

Although the sensory pathways of the bladder, glans penis, clitoris, and vagina are well characterized (1), the human urethra remains understudied. Far from being a simple conduit for the elimination of urine and semen, the urethra is a complex multilayered structure containing epithelial and neuroendocrine cells, vascular and muscular components and dense autonomic and somatic innervation (1), which allows coordinated actions with the bladder for both urination and ejaculation (2). Evidence suggests that specialized non-neuronal sensory cells detect mechanical and chemical stimuli such as urine flow, distension, osmolarity, and inflammation, thereby modulating afferent signaling to the CNS. Research in non-human mammals indicates that urethral afferents contribute to bladder reflexes and visceral sensation, including nociception, via neuronal activation and signaling at the spinal cord level (3). CNS-mediated urethrovesical circuits elicit and amplify bladder contractions and contribute to effective voiding. Conversely, blockade of urethral afferent signals, for instance, with sodium channel blockers such as lidocaine, attenuates bladder contractions induced by distension or urethral flow and impedes voiding (4). Here, cells of epithelial origin, specialized with a tuft of apical microvilli, so-called "brush cells", analogous to the cell specializations described in the respiratory and gastrointestinal tracts, can detect luminal contents, and initiate protective reflexes in response to potentially noxious substances (4). These sensory receptors have also been described in the prostatic urethra and ejaculatory ducts, along with microvilli, nerve plexuses and type A nerve fibers that may be involved in both sensory and transduction functions. Studies in animals have demonstrated the sensory response of these cells in the posterior urethra, suggesting a defensive role against injury such as bacterial damage, or their involvement in the bladder reflex response and voiding efficiency as short local neural subcircuits (1, 5, 6) Thus, free nerve endings detect sensory stimuli but, as nerves may not reach the lumen where most sensory input originates, it is likely that the urethral epithelial cells first detect stimuli from the cellular lining of the urethral lumen (5). It has also been shown that the urethra is densely innervated by sensory nerves positive for calcitonin gene-related peptide (CGRP) and substance P, as well as having specialized epithelial cells involved in sensing, processing, and transmitting sensory information (6, 7).

Neuroendocrine receptors can bind to either hormones or neurotransmitters, regulating cellular functions in various organs, including the urethra, where they mediate smooth muscle contraction and relaxation. Examples of neuroendocrine receptors include adrenergic receptors, acetylcholine-sensitive muscarinic receptors, serotonin receptors, and oxytocin receptors (2). In men, elevated prolactin and serotonin levels following ejaculation have been associated with transient erectile and ejaculatory inhibition (1). Oxytocin plays a paracrine role in stimulating the contractility of seminiferous tubules, the epididymis and prostate gland. It also modulates androgen levels in these tissues by enhancing the conversion of testosterone to dihydrotestosterone via 5α-reductase 2 (8). Transgender women often use estrogen therapy to develop certain female characteristics and suppress male characteristics (9, 10), with dissimilar effects on testicular tissue, possibly due to heterogeneous doses or treatment duration (11), although it has been shown to be effective and generally well tolerated (12). In contrast to studies of the bladder urothelium in relation to sensory afferents using histological, immunohistochemical and molecular techniques, little has been described in the human anterior urethra; most research has focused on the prostatic urethra or non-human mammals (13, 14). However, it has been demonstrated that certain processes observed in laboratory animals cannot be replicated in humans (15, 16). We hypothesized that the density of neuroendocrine cell in the penile urethra decreases with age but that this decrease may be prevented or even reversed by long-term estrogen therapy. The aim of this case series study was to determine the distribution of neuroendocrine cells in the penile urethra and to evaluate their association with age and the duration of estrogen therapy.

## MATERIALS AND METHODS

The present study protocol was reviewed and approved by the Institutional Review Board of the Carlos van Buren Hospital Directive and by the Scientific Ethics Committee SSVSA, Valparaiso Region (IRB approval number: 04-2020). In this retrospective descriptive immunohistochemical-based cohort study, we examined twenty-six pieces of inert biological material from the anterior urethra of males obtained during feminizing genitoplasty surgery in transsexual patients. The inclusion criteria were female transsexual patients over 18 years of age undergoing sex reassignment surgery. Exclusion criteria included previous genital reassignment surgery, traumatic urethras, and oncological urethras. All participants were recruited between 2020 and 2022 at the Department of Urology, Carlos van Buren Hospital, after providing written informed consent prior to surgery.

**Figure 1:**
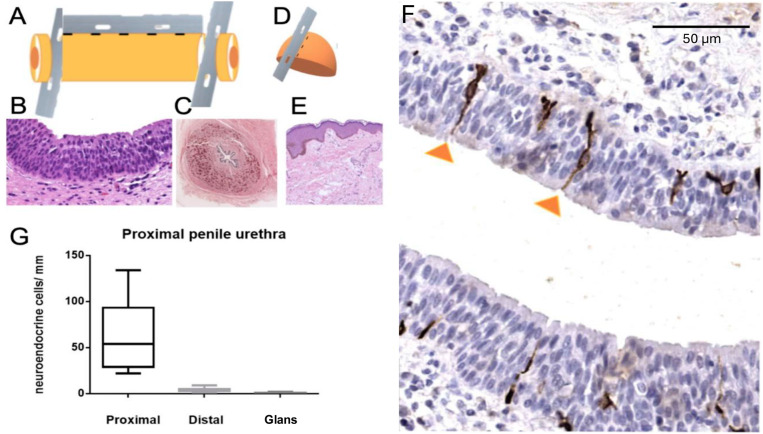
Detection of neuroendocrine cells in the penile urethra by double immunohistochemistry.

Clinical data were obtained from the medical records initiated at the start of feminization treatment in the same hospital department. There were no missing data in our sample. Penile urethras were collected fresh and fixed in 10% buffered formalin. For this study, we defined the proximal (bulbar) penile urethra as the segment located between the membranous urethra and the penoscrotal junction, and the distal penile urethra as the segment extending from the mid-shaft to the glans. The glans urethra was analyzed separately as its own region. Three cuts were thus made in each urethra for sample analysis: proximal penile urethra, distal penile urethra, and glans urethra (Figure-1A-D). Cell identification was performed by dual chromogenic sequential immunohistochemistry. Briefly, representative areas of each urethral section were identified by the team's pathologist. Control tissues used were skin, testis, pituitary adenoma, and cerebral cortex from our biorepository. Formalin-fixed and paraffin-embedded blocks were sectioned, and antigen retrieval was performed in a DAKO TP-Link with an alkaline bath. Tissue enzymes were blocked with EnVision FLEX PEROXIDASE-BLOCKING REAGENT (SM801) (17). DAB polymer chromogen and alkaline phosphatase were used for detection. Sequential double immunostaining was performed on a Leica Bond-Max auto-stainer according to the manufacturer's protocol. After antigen retrieval, slides were sequentially incubated with the primary antibody for β III tubulin and the secondary antibody against the neuroendocrine function markers chromogranin A or PGP9.5 (Table-1). Images were captured using a Leica DM2000 microscope with a 63x oil immersion objective. For each urethral section, three representative fields were evaluated and the area with the highest density of immunohistochemical staining was identified by the pathologist. The number of cells positive for chromogranin A was quantified. The total number of positive cells and the mean cell density, expressed as cells per mm^2^ of epithelium, were calculated. This was done using an automated cell counter in ImageJ software (NIH, Bethesda, MD). ImageJ allows a staining intensity threshold to be set, enabling it to automatically detect objects that meet that criterion and transform each positive cell into a counting point. The variables considered were age, years of estrogen use and neuroendocrine cell density in each urethral section. Quantitative variables were summarized as median and interquartile range. The urethral epithelium was quantitatively described by characterizing the number of positive cells per mm^2^ for each biomarker. Cells were classified by localization, morphology, polarity, and label intensity. Biomarker clustering of variables was analyzed using the k-means algorithm based on estrogen exposure. This enabled us to distinguish between two groups of patients based on the timing of estrogen exposure rather than age, which would have revealed a negative correlation with neuroendocrine cell density. Ultimately, we examined the association between the duration of estrogen exposure and the number of neuroendocrine cells in the bulbar urethra using linear regression.

(A) Diagram of the section of the proximal and distal penile urethra. Hematoxylin-eosin staining (B) Longitudinal view and (C) Transverse view of the penile urethra. (D) Glans section. (E) Side view of glans urethra. (F) Double immunohistochemistry for chromogranin A - DAB (brown +) and PGP9.5 (red -). (G) Density of neuroendocrine cells per mm of stretched urothelium. Arrows indicate notches typical of complete transepithelial cells. Scales are 100x.

**Table 1:** Tissue-specific proteins used as molecular markers to identify and differentiate human urethral tissues.

Protein	Description	Presence in Urethra
Anti-β III Tubulin antibody neuronal marker (TUBB3, 1:200, ab78078, Abcam)	Class III β-tubulin is one of the seven β-tubulin isotypes found in the human genome, neurons and testis.	β-III tubulin can be detected in the proximal urethra using immunolabeling techniques (23).
Anti-Chromogranin A antibody (Cg A, RTU, BSB5346BIOSB)	Chromogranin A (Cg A) is a protein that belongs to the granin family of neuroendocrine secretory proteins. It is found in secretory vesicles of neurons and endocrine cells.	Chromogranin A can be detected in urethras obtained from radical cystectomy specimens using immunolabeling techniques (20).
Rabbit polyclonal PGP9.5 antibody (UCHL1, 1:300, ab227157)	Protein gene product 9.5 (PGP 9.5) is a neuron-specific protein that has been used as a marker for neurons and neuroendocrine cells.	PGP9.5 is a general nerve marker that has been found to be associated with blood vessels, glands, and cells in the urethra (24).

## RESULTS

**Figure 2:**
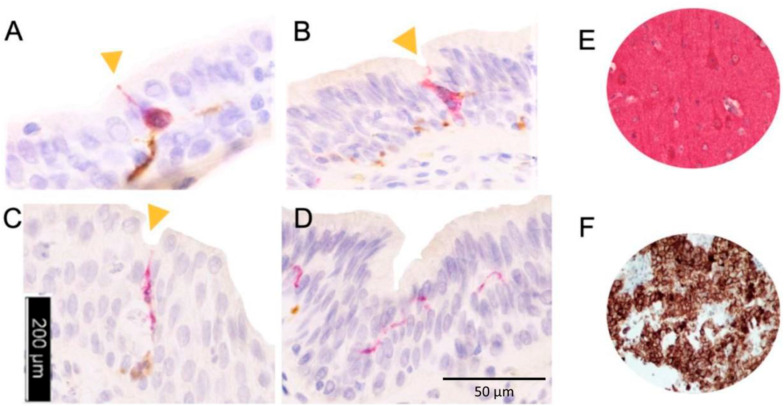
Evidence of urothelial paraneurons with neural projections reaching urethral lumen.

**Figure 3:**
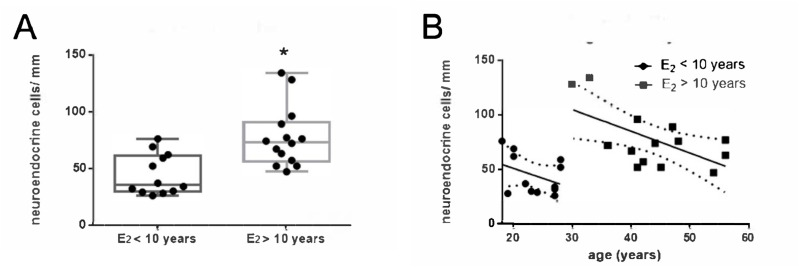
Neuroendocrine cell density and rate of decline by years of estrogen use.

The cohort comprised 26 patients. Group 1 (n=12) had a median age of 23.5 years (20-27) and median estrogen exposure of 5.5 years (4.8-6.2), while Group 2 (n=14) had a median age of 43.0 years (40-47.8) and median estrogen exposure of 18.0 years (18-22.2) (both p<0.001). Comorbidities, esthetic procedures, and life stage did not differ significantly between groups (Table-2). Representative histology, immunohistochemical staining, and quantitative associations are illustrated in Figures 1–3. Dual immunohistochemistry for chromogranin A positive and PGP9.5 negative (Figure-1E) allowed visualization and quantification (Figure-1F) of surface elongated, bipolar, transepithelial endocrine cells specifically concentrated in the bulbar urethra. A significant gradient of neuroendocrine cell density was observed, with the bulbar urethra showing the highest values compared to distal urethra and glans (Figure-1G). Co-expression of tubulin III and chromogranin A is shown in Figure-2. These neuroendocrine cells have dendrite-like processes that extend from the luminal to the basal layers of the epithelium (Figure-2A). Some have an apical tuft of microvilli on the luminal surface of the urethra, termed "open" neuroendocrine cells, and are thought to respond to luminal stimuli (Figure-2B) (2). The co-expression of tubulin III and chromogranin shows luminal and lateral polarity, respectively, with typical scission in transepithelial cells (Figure-2C). Nerve extensions were identifiable from the urothelium to the glans in transverse sections (Figure-2D).

**Table 2:** Demographic and clinical characteristics by k-means clustering defined groups (n=26).

Variable		Group 1 (n=12)	Group 2 (n=14)	p-value
**Age (years)**		23.6 ± 3.8; Median 23.5 (20-27)	43.8 ± 8.0; Median 43.0 (40-47.8)	<0.001
**Estrogen use (years)**		5.1 ± 2.5; Median 5.5 (4.8–6.2)	19.8 ± 5.5; Median 18.0 (18–22.2)	<0.001
**Comorbidities: None**		9 (75%)	11 (78.6%)	0.64 (Fisher)
	Comorbidities: ≥1	3 (25%)	2 (21.4%)	
**Esthetic procedures: None**		7 (58.3%)	10 (71.4%)	0.77 (Chi^2^)
	Esthetic procedures: Implant	4 (33.3%)	3 (21.4%)	
	Esthetic procedures: Toxin/Botox	1 (8.3%)	1 (7.1%)	
**Life stage: Childhood**		7 (58.3%)	9 (64.3%)	0.95 (Chi^2^)
	Life stage: Youth	3 (25%)	3 (21.4%)	
	Life stage: Adulthood	2 (16.7%)	2 (14.3%)	

Groups were defined according to estrogen exposure time. Variables were extracted from medical records and physical examination.

(A) Typical paraneurons in the urothelium (B) Open paraneuron in the urothelium (C) Transepithelial neuroendocrine cell in the urothelium (D) Nerve processes of the urothelium (E) bIII tubulin control of a temporal cortex (F) Chromogranin A control of a pituitary adenoma. Photographs are scaled 100x.

(A) k-means clustering algorithm in two groups with respect to neuroendocrine cell density in the proximal urethra and age (p <0.005). (B) Linear correlation by cluster between the number of neuroendocrine cells in the proximal urethra and the years of estrogen use.

Significantly higher bulbar neuroendocrine cell density in patients with >10 years of estrogen exposure compared with those with shorter exposure (Figure-3A) was identified with the Mann-Whitney k-means test (p < 0.005). The negative linear relationship observed between age and the number of neuroendocrine cells in the bulbar urethra was confirmed (Figure-3B).

## DISCUSSION

Neuroendocrine cells were predominantly concentrated in the bulbar urethra, showing a negative correlation with age and a positive association with prolonged estrogen exposure. These findings may be clinically relevant in male-to-female transition, as preservation of the bulbar urethra could enhance sensory function and sexual satisfaction. The lower urinary tract is richly innervated by parasympathetic, sympathetic, and somatic motor and sensory fibers carried by the pudendal nerves originating from the penile nerves, with efferences that contract the urethral sphincter and some populations of pudendal afferents that innervate the genital organs and the cutaneous and subcutaneous tissues of the perineum (4, 18). The afferent nerves are distributed between the muscle fibers, around blood vessels, in the urothelium, and in a dense sub-urothelial plexus. Some myelinated fibers not only get the striated muscular layer, but they reach the smooth muscle layer and the submucosa, suggesting that these fibers could correspond to sensory nerves (5). It has been described in some animal species that afferent nerves extend to the luminal surface of the urothelium (4). The bulbar urethra and the bladder neck concentrate the largest density of sensory neurons in the lower urinary tract, which can release a cascade of epithelial stimulatory or inhibitory mediators, with a key role in sensation (19) and probably in muscle tone. Thus, urethral afferents may be implied in generating pain sensation, but also in the sense of filling and the desire to micturate (3).

The neuroendocrine cells of the urothelium express numerous receptors and ion channels and secrete mediators that can activate or inhibit sensory neurons (19). Their observed polarity, with tubulin III localized luminally and chromogranin A laterally, suggests a specialized role in neurochemical modulation.

Besides neuroendocrine cells, several sensory structures have been identified in the urothelium, such as purinergic receptors, protease-activated receptors, mechanosensitive epithelial sodium channels, and transient receptor potential (TRP) ion channels (20), which allows the urothelium to respond to sensory stimuli mediated by substances such as adenosine, norepinephrine, ACh, bradykinin, neurotrophins, endothelin, and estrogens. Responses include increased stretch during bladder filling, release of soluble factors or chemical mediators, peptides and transmitters by nerves, inflammatory cells and even blood vessels (21, 22). Furthermore, the population of cells situated near afferent nerve fibers that contain nicotinic ACh receptors in the bladder urothelium express TRP, which are normally found in nerves (21). These cholinergic chemosensory cells release ACh in response to the presence of bitter chemicals, such as those produced during bacterial infection, with subsequent increase in bladder activity (22, 23). Urothelial neuroendocrine cells have been shown to release serotonin in several mammalian species (3). Although the role of 5-HT in the urethral epithelium has not been elucidated, since neuroendocrine cells can detect and transmit sensory information to the CNS, it is likely that 5-HT helps regulate muscle tone, urinary function, inflammation and the sensation of pain and pleasure in the penis by regulating the release of neuropeptides and neurotransmitters.

The neuroendocrine cell gradient reported here is consistent with reported studies in rodents, in which neuroendocrine cells were concentrated in the prostatic urethra and attached to CGRP-immunoreactive nerves; the authors speculated that these cells could release 5-HT in response to luminal stimuli, and that both neuroendocrine cells and CGRP-immunoreactive nerves could regulate each other by a reflex mechanism (6). Therefore, could these neuroendocrine cells correspond to a new class of chemosensory sentinel cells?

The study has limitations, including the retrospective design, which may have introduced uncontrolled confounders such as estrogen dosage, or concomitant medications, but it is unlikely that we could obtain urethras from healthy men or women to be used as controls. Deceased organ donation could help provide controls in future developments of this research. Eventually, the small sample size may have reduced statistical power. Nonetheless, significant differences were observed, emphasizing the need for further research on estrogen's impact on urethral neuroendocrine cell density.

Clinically, while most studies of hormone therapy in transgender patients have emphasized adverse effects, such as cardiovascular or thromboembolic events, osteoporosis (8), or body composition (9, 10), few studies have examined the sexual quality of life and most of the literature in this area has focused only on sexual function and not on sexual satisfaction (11). Since the advent of sex reassignment surgery, physicians have paid increasing attention to the preservation of sexual function. It is noteworthy that the results of sex reassignment surgery have shown great variability in male-to-female transsexuals, with orgasmic capacity ranging from 27 to 100% postoperatively (24). Our findings highlight the importance of preserving the bulbar urethra in male-to-female sex reassignment surgery, a consideration that may inform future refinements in surgical techniques and hormone treatment protocols.

Estrogen appears to regulate urethral muscle tone and sensitivity, while low estrogen levels are associated with increased risk of urinary tract infections and bladder dysfunction (25). Although aromatase expression in penile tissue, particularly in the corpus cavernosum, has been implicated in local estrogen modulation and erectile physiology (26), its role in the urethra remains unexplored. Both androgen and estrogen receptors are expressed in the urethral and bladder epithelium and smooth muscle (27), suggesting estrogen-specific effects on neurotransmission, epithelial protection, and inflammation. These mechanisms may influence urinary urgency, pain perception, and orgasmic intensity. Further research will help determining whether these findings are due to exogenous estrogen intake or to local androgen-to-estrogen aromatase-mediated conversion. Interestingly, the concentration of neuroendocrine cells and sensory structures in the proximal urethra may provide a biological basis for phenomena such as intra-urethral stimulation or masturbation, warranting further investigation.

## CONCLUSIONS

This study provides evidence of a gradient of neuroendocrine cells in the male urethra, concentrated in the bulbar region, and demonstrates that their density declines with age but can be preserved by long-term estrogen therapy. Clinically, these findings emphasize the bulbar urethra as a key sensory structure that should be preserved during genitoplasty and urological procedures. They also suggest that hormone therapy may mitigate age-related sensory decline, with implications for improving postoperative sexual function and urinary outcomes in transgender women and possibly in aging males. Further research may help elucidate the functional roles of different dose combinations and administration routes of androgens and estrogens in sexual tissues.

## Data Availability

Uninformed

## References

[1] Fowler CJ, Griffiths D, de Groat WC (2008). The neural control of micturition. Nat Rev Neurosci.

[2] Shih C, Cold CJ, Yang CC (2013). Cutaneous corpuscular receptors of the human glans clitoris: descriptive characteristics and comparison with the glans penis. J Sex Med.

[3] Kullmann FA, Chang HH, Gauthier C, McDonnell BM, Yeh JC, Clayton DR (2018). Serotonergic paraneurones in the female mouse urethral epithelium and their potential role in peripheral sensory information processing. Acta Physiol (Oxf).

[4] Gustafson KJ, Creasey GH, Grill WM (2004). A urethral afferent mediated excitatory bladder reflex exists in humans. Neurosci Lett.

[5] Deckmann K, Kummer W (2016). Chemosensory epithelial cells in the urethra: sentinels of the urinary tract. Histochem Cell Biol.

[6] Jung SY, Fraser MO, Ozawa H, Yokoyama O, Yoshiyama M, de Groat WC (1999). Urethral afferent nerve activity affects the micturition reflex: implication for the relationship between stress incontinence and detrusor instability. J Urol.

[7] Birder LA, de Wachter S, Gillespie J, Wyndaele JJ (2014). Urethral sensation: basic mechanisms and clinical expressions. Int J Urol.

[8] Shafik A, Shafik IA, El Sibai O, Shafik AA (2007). Effect of urethral stimulation on vesical contractile activity. Am J Med Sci.

[9] Glintborg D, T’Sjoen G, Ravn P, Andersen MS (2021). Management of endocrine disease: Optimal feminizing hormone treatment in transgender people. Eur J Endocrinol.

[10] Thackare H, Nicholson HD, Whittington K (2006). Oxytocin—its role in male reproduction and new potential therapeutic uses. Hum Reprod Update.

[11] Schneider F, Kliesch S, Schlatt S, Neuhaus N (2017). Andrology of male-to-female transsexuals: influence of cross-sex hormone therapy on testicular function. Andrology.

[12] Gorton RN, Erickson-Schroth L (2017). Hormonal and surgical treatment options for transgender men (female-to-male). Psychiatr Clin North Am.

[13] Aumüller G, Doll A, Wennemuth G, Dizeyi N, Abrahamsson PA, Wilhelm B (2012). Regional distribution of neuroendocrine cells in the urogenital duct system of the male rat. Prostate.

[14] Lendon RG, Dixon JS, Gosling JA (1976). The distribution of endocrine-like cells in the human male and female urethral epithelium. Experientia.

[15] Holstege G, Georgiadis JR, Paans AMJ, Meiners LC, van der Graaf FHCE, Reinders AATS (2003). Brain activation during human male ejaculation. J Neurosci.

[16] Le Moëne O, Ågmo A (2019). Modeling human sexual motivation in rodents: some caveats. Front Behav Neurosci.

[17] Suhaj P, Do D, Olejar T, Pichova R, Lang O, Matej R (2024). PPY-cell hyperplasia accompanying NENs: immunohistochemical and nuclear medicine analysis. Pathol Res Pract.

[18] Di Sant’Agnese PA, deKlerk DP, Jensen M (1983). Endocrine-paracrine (APUD) cells of the human female urethra and paraurethral ducts. Anat Rec.

[19] Andersson KE (2018). Urethral afferent signalling: role of 5-HT paraneurons. Acta Physiol (Oxf).

[20] Jen PY, Dixon JS, Gosling JA (1995). Immunohistochemical localization of neuromarkers and neuropeptides in human fetal and neonatal urinary bladder. Br J Urol.

[21] Beckel JM, Kanai A, Lee SJ, de Groat WC, Birder LA (2006). Expression of functional nicotinic acetylcholine receptors in rat urinary bladder epithelial cells. Am J Physiol Renal Physiol.

[22] Apodaca G (2004). The uroepithelium: not just a passive barrier. Traffic.

[23] Perniss A, Schmidt P, Soultanova A, Papadakis T, Dahlke K, Voigt A (2021). Development of epithelial cholinergic chemosensory cells of the urethra and trachea of mice. Cell Tissue Res.

[24] Klein C, Gorzalka BB (2009). Sexual functioning in transsexuals following hormone therapy and genital surgery: a review. J Sex Med.

[25] Lüthje P, Hirschberg AL, Brauner A (2014). Estrogenic action on innate defense mechanisms in the urinary tract. Maturitas.

[26] Schulster M, Bernie AM, Ramasamy R (2016). The role of estradiol in male reproductive function. Asian J Androl.

[27] Rosenzweig BA, Bolina PS, Birch L, Moran C, Marcovici I, Prins GS (1995). Location and concentration of estrogen, progesterone, and androgen receptors in the bladder and urethra of the rabbit. Neurourol Urodyn.

